# Wearable Intrinsically Soft, Stretchable, Flexible Devices for Memories and Computing

**DOI:** 10.3390/s18020367

**Published:** 2018-01-27

**Authors:** Krishna Rajan, Erik Garofalo, Alessandro Chiolerio

**Affiliations:** 1 Istituto Italiano di Tecnologia, Center for Sustainable Future Technologies, Corso Trento 21, 10129 Torino, Italy; krishnarajan.krishnarajan@studenti.polito.it (K.R.); g.erik1992@gmail.com (E.G.); 2 Department of Applied Science and Technology, Politecnico di Torino, Corso Duca degli Abruzzi 24, 10129 Torino, Italy

**Keywords:** e-textiles, Wearable Memories &amp, Computing Devices, resistive switching devices, memristors, wearable, flexible, stretchable, soft electronics, knitted electronics, printed electronics

## Abstract

A recent trend in the development of high mass consumption electron devices is towards electronic textiles (e-textiles), smart wearable devices, smart clothes, and flexible or printable electronics. Intrinsically soft, stretchable, flexible, Wearable Memories and Computing devices (WMCs) bring us closer to sci-fi scenarios, where future electronic systems are totally integrated in our everyday outfits and help us in achieving a higher comfort level, interacting for us with other digital devices such as smartphones and domotics, or with analog devices, such as our brain/peripheral nervous system. WMC will enable each of us to contribute to open and big data systems as individual nodes, providing real-time information about physical and environmental parameters (including air pollution monitoring, sound and light pollution, chemical or radioactive fallout alert, network availability, and so on). Furthermore, WMC could be directly connected to human brain and enable extremely fast operation and unprecedented interface complexity, directly mapping the continuous states available to biological systems. This review focuses on recent advances in nanotechnology and materials science and pays particular attention to any result and promising technology to enable intrinsically soft, stretchable, flexible WMC.

## 1. Introduction

“Flexible”, “Stretchable”, “Bendable” and “Wearable” are some of the most common terminologies which may dramatically change our individual interaction scheme without impacting on the way of living in the very near future. This will occur by enabling a more natural interaction between humans and electron devices, shifting the paradigm from the actual “User”, somebody that physically interacts voluntarily with a physical object such as a smartphone or a computer, to the next evolutionary step, the “I-user”, somebody that physically interacts involuntarily by simply wearing her/his outfit (physically unbound from the user’s body, yet connected to it by means of non-invasive electrodes). With an ultimate effort, the last evolutionary step will make us “A-user”, augmented users that integrate new or augmented biological functionalities through bio-compatible integrated electron devices (physically implanted in the user’s body).

Research in field of stretchable and bendable electronics is thriving day by day in the field of smart textiles or electronic textiles (e-textiles) [1]. In the present scenario, these flexible electronic devices are in high demand from the medical industry [2], for real-time patient monitoring. Nonetheless, from the most technical and specific usage to the most common and private one, smart wearables have the potentiality to fill the gap between standard clothes and electronics even for the most reluctant.

We here introduce the terminology Wearable Memories and Computing devices (WMC) to create a distinction between this area, where each component is meant to be fabricated with innovative technologies and novel materials, and the Wearable Computers, known since the 1970s, integrated into clothes using the basic discrete electronics components encased and adapted to textiles. The former topic is dealt here, while the latter is not object of our treatise.

Wearable Memories and Computing devices (WMC) will enable each of us to contribute to open and big data systems as individual nodes, providing extremely valuable real-time and super fine granularity information about physical and environmental parameters. Such data are of primary importance, and include air pollution monitoring, sound and light pollution, chemical or radioactive fallout alert, network availability, and so on. Furthermore, WMC could be directly connected to human brain [3] and enable extremely fast operation and unprecedented interface complexity, directly mapping the continuous states available to biological systems. We strongly believe that a real, feasible, reasonable advancement towards cybernetics, smart prosthesis and life augmentation, goes beyond the use of both standard Complementary Metal Oxide Semiconductor (CMOS) circuits and CMOS based neuromorphic emulators. Literature already reports several promising and interesting results regarding totally printed Thin Film Transistors (TFTs) (e.g., [4,5,6]), as well as other key enabling technologies, such as printable conductors to realize electrical connections and transfer energy or signal across a textile [7,8]. Additive/digital manufacturing appear suited for the development of really flexible, stretchable, wearable devices, while standard, solid-state electronics still will need a (weak, brittle) connection to the real macroscopic world.

However, the present review delves into the development of resistive switching devices (RSD), otherwise known as memristors, as the most promising candidates towards WMC applications, including their potentiality for neuromorphic computing and direct coupling with biological brains. This conclusion is supported by several authors, to cite one, in an extremely synthetic and logic reasoning [9]: (1) a brain-inspired computing system should ideally employ some form of non-volatile memory; and (2) the dominant non-volatile technology, flash, is expected to be superseded by novel technologies, such as Phase Change Memory (PCM), spin transfer torque random access memory (STT-RAM) or resistive random access memory (ReRAM).

**Figure 1:**
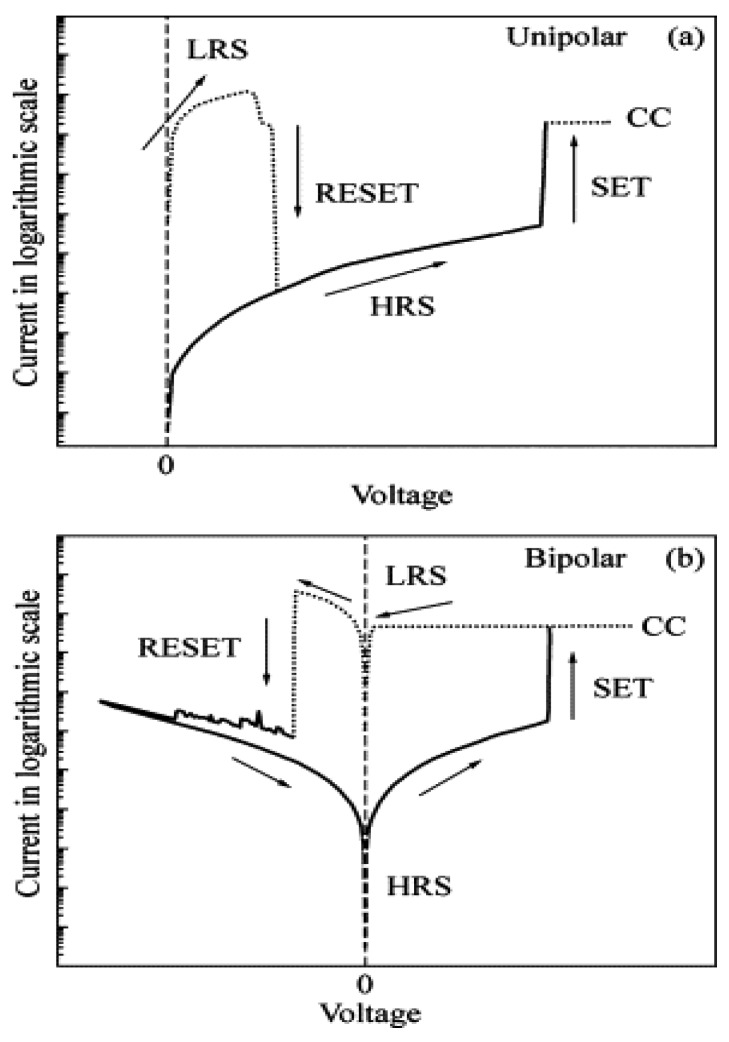
I-V curves of: (**a**) unipolar switching; and (**b**) bipolar switching [16] (with permission from Elsevier).

RSDs (PCM + STT-RAM + ReRAM + other technologies not yet commercial), theoretically predicted over forty years ago [10] and experimentally studied for a decade [11], are among the few emerging technologies that catalyzed unprecedented worldwide attention due to a variety of applications, including instant turn-on computers, analog memories with continuum states for learning machines, nanoscale memristive synapses [12], and so on. Device speed, scalability and the ease of fabrication are some of their features [13]. RSDs have two or more discrete resistive states: in the former case, the high resistance state (HRS) corresponds to the Off state and the low resistance state (LRS) corresponds to the On state; in the latter, apart from the high and low resistance states, it also has intermediate resistance states (IRS) [14,15]. By applying the apt set voltages, the device can be moved from HRS through IRS to LRS. Multilevel RSDs are the most preferred for multilevel storage, thus enhancing the storage density without much change in the technology [15]. An RSD or a memristor can switch between these states by the application of an appropriate electric stimulus. The transition from Off to On state is known as Set and the transition from On to Off state is known as Reset. The voltage at which the device moves from Off to On is known as the set voltage and the voltage at which the device moves from Off to On state is known as the reset voltage. RSD is said to be non-volatile based on its ability to retain its past resistance state, after the electrical stress is removed; therefore, it represents the best solution for low energy permanent memories. Switching between states can be classified into unipolar and bipolar. The unipolar switching occurs when the reset takes place at a higher current and at a voltage below the set voltage (Figure 1a), while the bipolar switching occurs when the set and reset take place at two different polarities (Figure 1b) [16].

The advantages of using RSDs as storage class memories are: small feature size, lower power requirements, higher memory density, and its advantages go on [16,17,18,19,20,21,22,23,24,25,26,27].

The final goal in the field of RSDs is to physically implement an artificial neural network (ANN) for artificial intelligence (AI) purposes [22,24,28,29,30,31], through the emulation of biological synapses and biological phenomena such as spike-timing-dependent plasticity (STDP), a mechanism providing enhancement or depression of a specific communication channel based on the synchronization of pulses of the pre-synaptic and post-synaptic neurons [32]. Enhancing or depressing a response corresponds intuitively to storing or cancelling an information, respectively. We may say that “neurons that fire together wire together” [33], and add that musical instruments that do not play well in time are silenced by the director. In a similar way, the frequency of propagating pulses in a neuromorphic circuit are shifted by a variable time phase (that could be positive or negative) producing qualitatively a similar programming effect. We refer to an extremely detailed review (and references therein) for a complete screening of the device level engineering, showing how to implement such functionalities using available materials and processes [33].

In the following sections, we have classified literature in four main sections, highlighting the emergent properties fundamental for WMC applications: flexible, bio-based and biodegradable, stretchable and threaded devices. Of course, stretchable devices are also flexible. Optical transparency has not been considered, as this feature is not important for standard wearables, whose opacity guarantees everybody’s privacy—and, in the case of emergency, eventually safety and visibility. However, we should mention the importance of transparency for military applications, as appointed in the Emerging Security Technologies field [34], but this is outside the scope of the present review.

Regarding the testing of flexible and stretchable of wearable devices, we refer to specific literature [35,36].

## 2. Flexible RSDs for WMC

Flexible and printed electronics represent the new horizon for mass consumption electron devices industry, taking advantage of cheap substrates such as polymers and papers (both natural and nanoengineered) as well as fast and environmentally friendly additive fabrication technologies, already well settled, as analog (and digital) printing [37]. A few studies already focused on the development of printable inks enabling resistive switching [38,39]. Memristors or RSDs have also been developed on flexible substrates, using non-toxic, environmentally friendly materials [38]. The purpose of this applied research is not only towards smart wearable electronics or e-textiles, but also for complicated circuits where the production constraints limit the serviceability of rigid printed circuit boards (PCBs).

Yeom et al. [40] designed a transparent and flexible RSD that showed excellent memory performance even in its bent state. IZO (Indium Zinc Oxide) transparent electrodes (250 nm in thickness) were sputtered onto a flexible PET substrate. Al_2_O_3_ was then sputtered (RF) onto the IZO. The IZO top electrode was deposited vertically to form a crossbar structure. The transparent device had a transmittance of nearly 80% in the visible region (400–800 nm). It showed a transport behavior well described by Ohmic conduction [40] in the LRS and Poole–Frenkel emission model in the HRS [41]. The basic IV features a Set taking place in the negative polarity and the Reset taking place in the positive polarity. The reliability of an RSD is always determined in terms of its endurance and retention. The transparent flexible device prepared in the present case was found to have an excellent working endurance and retention at normal as well as in the bend state (10 mm bending radius).

## 3. Biobased/Biocompatible RSDs for WMC

**Figure 2:**
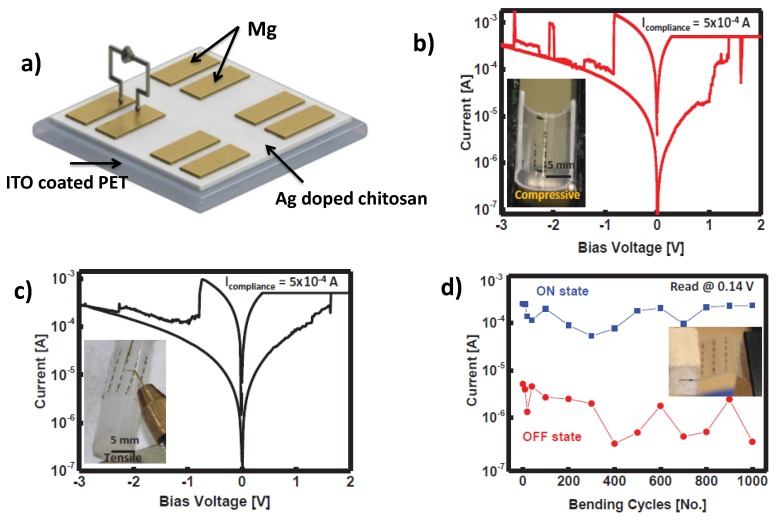
(**a**) Pictorial representation of the Mg/Ag doped Chitosan/Mg-RSD. Resistive switching behavior of the device after bending with a radius of curvature of 5 mm under: (**b**) Compressive stress; and (**c**) tensile stress. (**d**) Cyclic test performed at 0.14 V read voltage showing the stability of the RSD in its bend state [44] (with permission from Advanced Functional Materials).

Hosseini et al. [42] came up with flexible coplanar RSDs showing promising non-volatile memory applications. The major advantage of this RSD other than its flexibility is that it is a biocompatible device. Recent research has been carried out in the field of biodegradable electronics which can be implanted into human body and then be bio-adsorbed [43,44,45,46,47]. Here, a coplanar structure was designed using Mg electrodes and the switching matrix was fabricated by Ag-doped chitosan. Figure 2a gives the pictorial representation of the RSD. Mg has the outstanding properties of biodegradability and electrical conductivity. Chitosan on the other hand is an edible natural material [48,49]. The Ag-doped RSD exhibited non-volatile switching, which can be attributed to trap-related space-charge-limited conduction in high resistance state and filamentary conduction in low resistance state [15,50,51,52]. Bendability and performance under tensile and compressive stresses were evaluated. The IV features of the device under compressive and tensile stress are given in Figure 2b,c. The device was bent under the extremely small radius of 5 mm. The device maintained a stable On and Off current for consecutive 1000 cycles at a read voltage of 0.14 V, on mechanical flexibility tests with 5 mm radius of curvature (Figure 2d).

**Figure 3:**
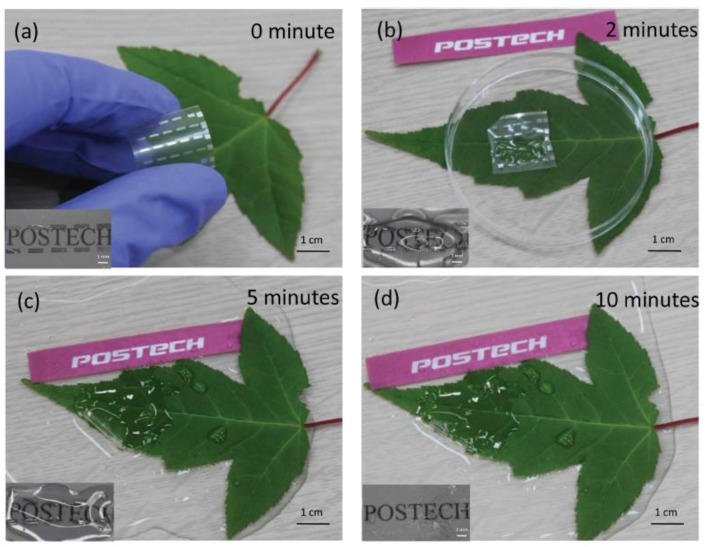
Decomposition of the Chitosan (substrate) and Mg (coplanar electrode) based RSD after dropping in water: (**a**) immediately dropped; (**b**) device after 2 min in water; (**c**) after 5 min; and (**d**) after 10 min [44] (with permission from Advanced Functional Materials).

It was also found that, by replacing the substrate with chitosan, and by using Mg as electrodes, the device could be completely decomposed. The Mg electrodes decomposed by reacting with water while the chitosan substrate also vanished by absorbing water (Figure 3a–d).

Other flexible RSDs have also been developed using other non-toxic, biocompatible, printable materials which include cellulose [53], egg albumen [54], *Aloe vera* film [55], silk protein [56], organic materials [57,58,59], etc.

## 4. Stretchable RSDs

**Figure 4:**
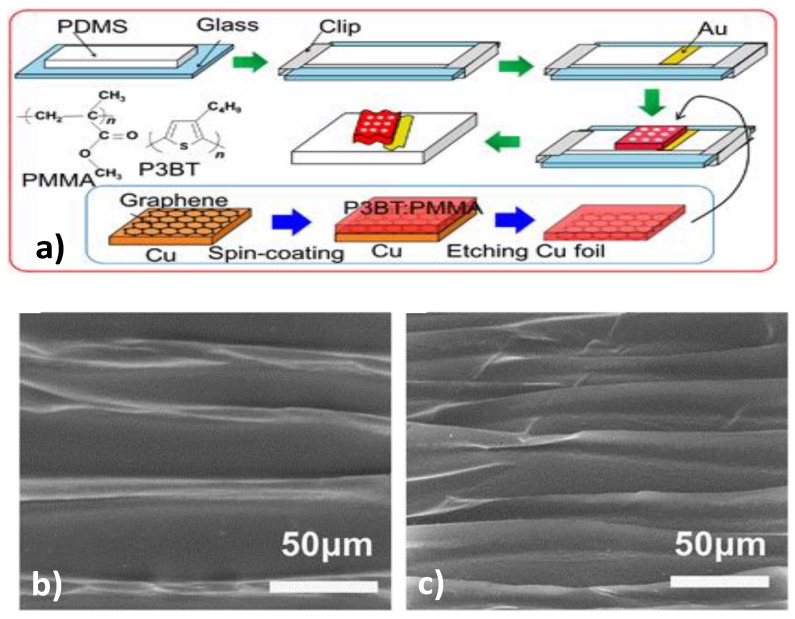
(**a**) Schematic representation of the Fabrication process for the stretchable organic memory devices, along with the chemical structures of PMMA and P3BT. PDMS; (**b**) SEM image of top view of rippled organic memory film; and (**c**) SEM image of angled view of rippled organic memory film [60] (with permission from Nature Publishing Group).

Stretchability is a fundamental mechanical feature that a device has to possess when it is knitted or glued to a stretchable band, or when it is directly coupled with human skin. There have been past studies using inorganic materials to come up with high performing stretchable and foldable integrated circuits. Lai et al. [60] designed a stretchable organic digital information storage device, which potentially advances the development of future smart and digital electronic system. One of the major advantages of this device is that it is a memory based on wrinkled structure, which takes advantage of elasticity and flexibility of organic materials and graphene [12]. The schematic process involved in the fabrication of the stretchable RSD is shown in Figure 4.

The blending solution of PMMA (PolyMethyl MethAcrylate) and P3BT (Poly 3-ButylThiophene) is spincoated onto the CVD (Chemical Vapor Deposition) deposited graphene/Cu foil (Supporting layer, Figure 4a). After etching away the Cu foil, the graphene/PMMA-P3BT membrane is then transferred onto a pre-stretched (50%) PDMS (polydimethylsiloxane) substrate and clamped onto a glass slide. To get contact with the bottom graphene, a gold layer was deposited onto the edge of the PDMS and it had partial contact with the graphene electrode. After the transferred film dried, the clamps were removed and the device was separated from the glass. The device (now film) spontaneously contracted and formed a ripple on the released substrate. Figure 4b,c shows the top view and angled view SEM image of the resultant rippled film.

**Figure 5:**
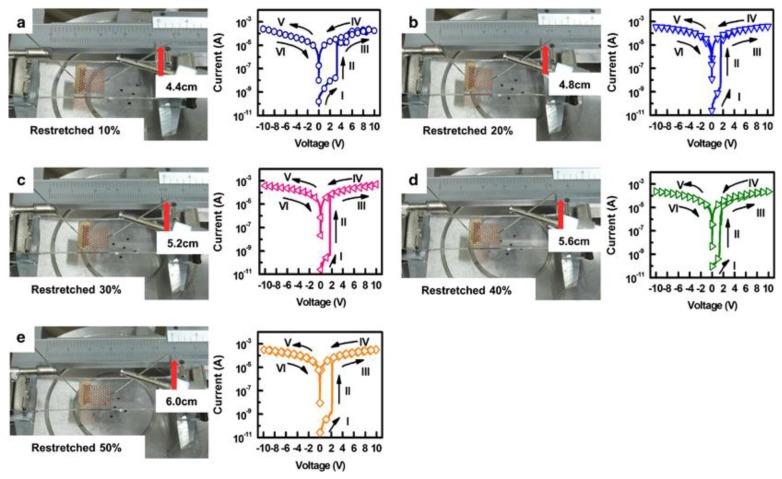
(**a**–**e**) Electrical switching characteristics on the RSD under different stretching condition (10% to 50%) [60] (with permission from Nature Publishing Group).

The electrical switching of the memory device at different stretching conditions were recorded (Figure 5). It was observed that the device under all conditions showed a Write Once Read many Memory (WORM) behavior [36,57].

Khiat et al. described a method which successfully transfer the Pt/TiO_2_/Pt based stacked RSD onto a flexible Parylene-C substrate. When electrically characterized, these flexible devices were able to exhibit both digital and analog memory obtained by the proper adjustment of the input pulsating scheme [61].

The complete device was a 32 × 32 array of single cells. This was peeled off a rigid Si wafer. The flexibility and stretchability of the device allowed the authors to roll the entire array of RSDs onto a finger. The device showed a non-volatile behavior with a pinched hysteresis loop. The RSD was bent and then later put onto a flat surface to achieve stable electrical contact to perform post bending tests. After bending stress, the RSD was still able to work properly for 8000 cycles even after the peeling-re-flattening process. To compare this result, we also have the endurance test performed on the device on a hard Si wafer which shows completion of 5000 cycles.

## 5. Threaded RSDs

The ultimate development of WMC is that of possessing directly threads that show the functionality of a memory or a computation element (or, even better, a logic gate).

**Figure 6:**
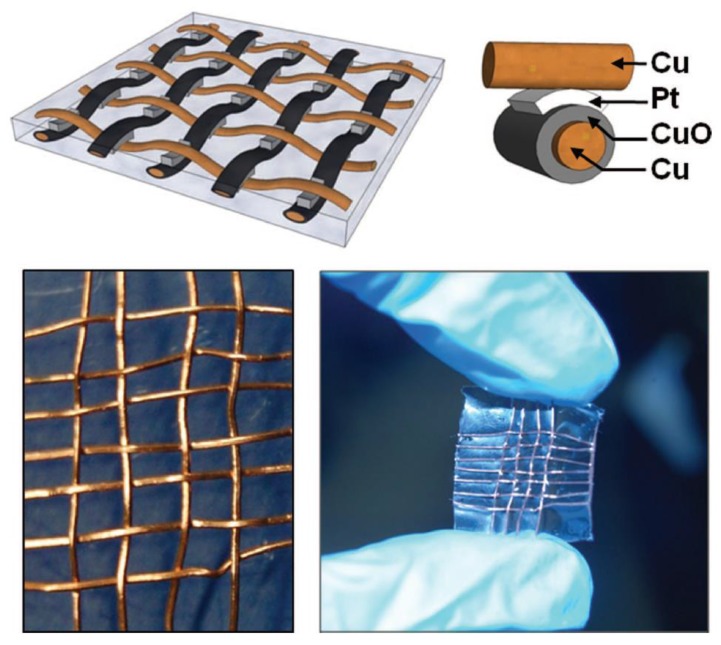
First full metal body threaded memory developed at NASA Ames Research Center in 2011 based on CuO and Pt [62] (with permission from the authors).

The first step into the field of threaded memories was performed at NASA Ames Research Center some years ago. Han and Meyyappan developed a full body metal threaded memory (Figure 6), taking advantage of the resistive switching showed by copper oxide when placed into contact with a noble metal such as Pt [62]. The functional properties were not astonishing, as well as mechanical flexibility, and a supposed very poor wearability.

**Figure 7:**
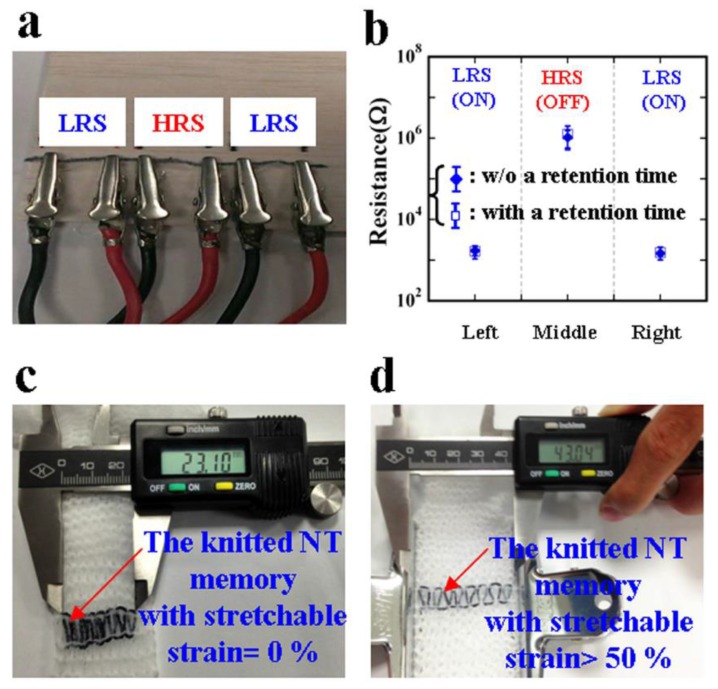
Threaded memory based on PEDOT:PSS and graphene nanocomposite nylon thread. (**a**) optical image showing three elements connected in series, having different resistive state; (**b**) electrical readout of their states; (**c**) un-strained knitted memory; (**d**) strained knitted memory [63] (with permission from the authors).

Kang developed a nylon threaded memory by exploiting a simple dip-and-dry process using a graphene–poly(3,4-ethylenedioxythiophene) polystyrene sulfonate (PEDOT:PSS) ink and the creation of a highly stretchable non-volatile memory [63]. The threaded memory so-fabricated appears to have typical write-once-read-many (WORM) feature (Figure 7).

**Figure 8:**
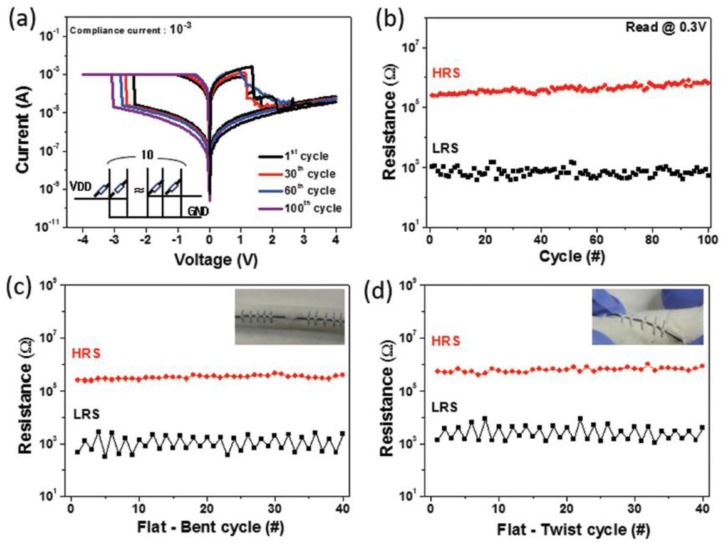
Threaded memory based on carbon thread and alumina wires, tested under mechanical load (bending and twisting) when arranged in an array of 10 devices linked together. (**a**) consecutive IV cycles, from #1 to #100; (**b**) HRS and LRS stability over 100 cycles; (**c**) HRS and LRS in the bent state; (**d**) HRS and LRS in the twisted state [64] (with permission from John Wiley and Sons).

Independently, and with a rather different approach, where we still see a carbon-based material, this time coupled with a metal oxide (Al_2_O_3_), Jo et al. created a real knitted Rewritable Random Access Memory (RRAM) [64]. The realization of such component requires the cross-knitting of the carbon-based and oxide-based wires and features outstanding properties, such as an On/Off ratio of 1000 stable over 1000 cycles, a retention time of at least 10,000 s, showing a mechanical stability of the response over at least 30 cycles, as well as small changes after a washing cycle (Figure 8).

## 6. Towards Wearable RSDs

Wearable electronic devices have seen great development in recent years and will be perhaps ready for the go-to-market in the next year. The main question that may arise in the reader could be: “What for?”. There is no specific demand for logic devices, other than components that are already much more easily integrated in a smartphone, in unparalleled numbers, with a considerable energy available and huge channels for data transfer. Of course, we must consider that technology development often takes routes that have not been announced before, and it is our opinion that the starting point should be found in a renovation of the garments. High-tech garments, or garments 2.0, will integrate body or environmental sensors, and it is there that we need logics, to process information and give a feedback to the user.

The Cu based threaded RSD demonstrated by Han and Meyyappan [62] and aluminum wire and carbon thread based threaded memory by Jo et al. [64] can both be considered as promising building blocks for future e-textiles.

Cai et al. presented a parylene based organic RSD which is very much suitable for WMC applications [65]. The device exhibits a high memory window of around 10^4^, an outstanding retention and great mechanical and electrical stability. Some important features of parylene include being a Food and Drug Administration (FDA) approved material that is safe for any implantation within the body. Furthermore, the device can be fabricated through a fully CMOS compatible process. The parylene based RSD enforces a sandwich structure of Al/parylene/W on a flexible substrate. The resistance switching is imparted by the 40 nm parylene layer and is transparent and flexible. Bending tests were performed to test its switching properties under bend circumstances. The HRS and the LRS at bent states were recorded and compared. The device was operated under three different conditions (10 mm, 20 mm and 30 mm bending radii). There was a small variation in the resistive states at various radii, indicating a superior bending stability and electrical reliability.

Li and Sun [66] reported the design, the experimental validation and application of a scalable, wearable e-textile triboelectric energy harvesting (WearETE) system for scavenging energy from activities of daily living. The WearETE system features ultra-low-cost material and manufacturing methods, high accessibility, and high feasibility for powering wearable sensors and electronics. Foam and e-textile are employed as active tribomaterials for energy harvesting and for their flexibility and wearability. Their results show the possibility of powering wearable electronics during human motion.

Another example concerns sports garments, in which functional properties have become crucial as well as comfort properties, since they improve the wearer performance. For this reason, Baskan et al. [67] designed and produced a sports vest and shorts, having high elastic recovery with fall detection sensor, by using flat-bed knitting machine. Several instrumental tests were conducted to verify comfort properties, tensile strength of elastomeric yarn, air permeability, moisture management, drape and objective handle (FAST tests) of garments. In this way, it was proven that shorts and vest samples have good comfort properties as a functional sport garment.

Regarding biomedical applications, because of their complexity, traditional force plates for postural stability assessment are unlikely applicable outside clinical environment; therefore, D’Addio et al. [68] evaluated the ability of new e-textile and wireless wearable sensor technologies to extend the posturography in new low-cost and home assessment contexts. Preliminary results showed a significant agreement between SF and the reference sensor measurements, suggesting a clinical use of Sensoria for low cost home care-based balance impairment assessments.

In addition, Zheng et al. [69] developed an armband wearable pulse transit time (PTT) system for 24-h cuff-less blood pressure measurement. They concluded that “the proposed wearable system has great potential to be used for overnight SBP monitoring, especially to measure the averaged SBP over a long period”.

Lou et al. [70] reported for the first time the fabrication of a self-assembled 3D film platform that combines a natural viscoelastic property material P(VDF-TrFe) (Poly[VinyliDeneFluoride-co-TriFluoroEthylene]) with an electrically conductive material reduced-Graphene Oxide (rGO) to fabricate high sensitive pressure sensors for applications as e-skins and wearable electronics. This device could be used also to monitor human physiological signals, ranging from blood pressure pulse to muscle movement, and the technology used in the future could be valuable for a range of applications of future wearable electronics for the prosthetic skins, sickness prevention and human–machine interfacing devices [70].

Other examples include the application of Wang et al. [71] related to an ultrasensitive cellular fluorocarbon piezoelectric pressure sensor (FPS) via a three-step hot-pressing method. They obtained tremendous piezoelectric activity by constructing micron sized voids in the inner cell, and combination with outstanding charge storage ability of the fluorocarbon electrets. The flexible and self-powered FPS owns the capability for detecting human motions such as wrist stretching, cheek motion from open/bite/open, eyes blinking and chest respiration, respectively. It can be also used for monitoring human physiological signals such as radial artery pulse wave. Moreover, Chen et al. [72] used a fiber-shaped textile, knitted with hierarchical polyurethane (PU) fibers, to fabricate a multifunctional e-textile by coating of silver nanowires (Ag NWs) and styrene–butadiene–styrene. Due to the creation of an Ag NWs conductive network, the inherent stretchability of PU fibers, and the hierarchical structure, the as-prepared e-textile exhibits high conductivity, high stretchability, high sensitivity, and multi-detection of strain and pressure. Moreover, the fabricated multifunctional e-textiles are also successfully weaved into electronic fabric for 2D force mapping.

Lanatà et al. [73] evaluated the performances of different wearable systems based on indirect breathing monitoring in terms of susceptibility to motion artifacts. Valenza et al. [74] investigated the possibility of using Electrodermal Response, acquired by a sensing fabric glove with embedded textile electrodes, as reliable means for emotion recognition. Finally, Li et al. [75] introduced a novel wearable sensor in intelligent clothing for human body temperature measurement based on optical fiber Bragg grating.

In addition, Cai et al. [76] demonstrated a nanophotonic structure textile with tailored infrared (IR) property for passive personal heating using nanoporous metallized polyethylene by constructing an IR-reflective layer on an IR-transparent layer with embedded nanopores to heat the immediate environment around the human body and save the energy wasted for heating the empty space of an entire building.

Moving toward other types of application, Yun et al. [77] reported e-textile gas sensors based on RGO coated on the commercially available yarns treated with Bovine Serum Albumin (BSA) as a molecular glue. The e-textiles show sensitive responses to NO_2_ and durability during cycles of bending stresses and washing treatments. Their study reports an ultrasensitive response of an e-textile to NO_2_ using combined sensing materials of transition metal disulfide (TMD) and RGO and they believe that the e-textile based gas sensors could be useful in wearable electronics applications requiring high sensitivity and excellent durability.

Other types of examples include the application developed by Zhou et al. [78]: they built a wearable textile-based humidity sensor for the first time using high strength and ultra-tough SWCNT (Single Wall Carbon NanoTube)/PVA (Polyvinyl Alcohol) filaments via a wet-spinning process. The particularity is that conductive SWCNT networks in the filaments can be modulated by adjusting the inter-tube distance by swelling the PVA molecular chains via the absorption of water molecules. Their smart textiles could pave a new way for the design of novel wearable sensors for monitoring blood leakage, sweat, and underwear wetting. Moradi et al. [79] created miniature implantable and wearable on-body antennas based on electrically conductive textiles and threads constituting an important milestone in the development of wireless brain–machine interface systems, and a new era of wireless body-centric systems.

Finally, funded by the European Commission, Curone et al. [80] developed a new generation garments for emergency-disaster personnel for continuously monitor risks endangering rescuers’ lives: the called Proe-TEX (Protection e-textiles: Micro-Nano-Structured fiber systems for Emergency-Disaster Wear).

Most wearable electronic devices that are currently available rely on rigid electronic components mounted on plastic, rubber or textiles. The final integration of all parts into a single device into the right form factor which can be finally incorporated into textiles or any such platform for the preparation of a wearable soft device is still a major hurdle. Another key problem the wearable industry is facing is the problem of power and state. Ultra-low voltage helps but true non-volatile memory, with the ability to perform simple logic-on-chip, is one of the threshold milestones to achieve [81].

The advancement in printing technology is a game changer in this context. The printed devices become flexible, washable and moreover require low power for applications in wearable electronics. Hybrid 3D printing is now becoming an important method for producing soft electronics [82]. This method involves the direct ink writing of conductive and dielectric elastomeric materials with automated pick-and-place of surface mount electronic components within an integrated additive manufacturing platform. This approach enables the printing of an insulating matrix and the conductive electrode inks onto specific layouts and finally print the conductive interconnects the complete electronic circuitry [82].

Nevertheless, in the future of wearable devices, we think that a combination of multiple techniques could provide the best compromise and more stable solution towards the go-to-market, for example hybrid printing together with threaded functionalities/sewing/knitting.

## 7. Conclusions

The primary use of any embodiment of RSDs as non-volatile memory and finally as neuromorphic devices makes this field interesting and technologically challenging for the integrated circuit industry, to expand in fields such as medicine. The vision of wearable memory and computing machines, in the sense of e-textiles, describes the future electronic systems for smart personal assistance, augmentation and recovery.

In the present review, we have tried to go through some important discoveries which report flexible, bendable and, some of them, even stretchable and biodegradable devices that have important future prospects to be used in wearable or even implantable devices. We do believe that epochal changes occur following a brand new route, unexpectedly. WMCs do not need to follow the same story of integrated electronics, therefore rely on solid state, opaque/rigid devices, in particular integrated circuits. Whenever the computational demand of a specific task assigned to WMCs would be outside its capabilities, the integrated wearable system could use the cloud for high performance real-time computing/storage (as happens for smartphones during complex tasks such as speech or image recognition).
